# Enhancing Evanescent Wave Coupling of Near-Surface Waveguides with Plasmonic Nanoparticles

**DOI:** 10.3390/s23083945

**Published:** 2023-04-13

**Authors:** Jerome Lapointe, Alexandre Grégoire, Jean-Philippe Bérubé, Réal Vallée

**Affiliations:** 1 Centre d’Optique, Photonique et Laser, Université Laval, 2375 Rue de la Terrasse, Québec, QC G1V 0A6, Canada; alexandre.gregoire.1@ulaval.ca (A.G.); rvallee@copl.ulaval.ca (R.V.); 2 Département de Chimie, Université Laval, 2375 Rue de la Terrasse, Québec, QC G1V 0A6, Canada; 3 Ciena Corporation, 505 Boulevard du Parc Technologique, Suite 100, Québec, QC G1P 4S9, Canada; jberube@ciena.com

**Keywords:** evanescent wave sensor, surface waveguide, plasmonic nanoparticles, femtosecond laser writing, PDMS stamp technique

## Abstract

Evanescent field excitation is a powerful means to achieve a high surface-to-bulk signal ratio for bioimaging and sensing applications. However, standard evanescent wave techniques such as TIRF and SNOM require complex microscopy setups. Additionally, the precise positioning of the source relative to the analytes of interest is required, as the evanescent wave is critically distance-dependent. In this work, we present a detailed investigation of evanescent field excitation of near-surface waveguides written using femtosecond laser in glass. We studied the waveguide-to-surface distance and refractive index change to attain a high coupling efficiency between evanescent waves and organic fluorophores. First, our study demonstrated a reduction in sensing efficiency for waveguides written at their minimum distance to the surface without ablation as the refractive index contrast of the waveguide increased. While this result was anticipated, it had not been previously demonstrated in the literature. Moreover, we found that fluorescence excitation by waveguides can be enhanced using plasmonic silver nanoparticles. The nanoparticles were also organized in linear assemblies, perpendicular to the waveguide, with a wrinkled PDMS stamp technique, which resulted in an excitation enhancement of over 20 times compared to the setup without nanoparticles.

## 1. Introduction

In recent years, optical waveguides have emerged as excellent tools to confine light in small structures and bring it to sites inaccessible to conventional spectroscopy. Several physical techniques such as thin film deposition by magnetron sputtering [1], ion-beam sputtering [2] or thermal oxidation [3], photolithography by e-beam evaporation [4] and pulsed laser deposition [5] have been developed for waveguide fabrication. Chemical techniques including gas phase processes such as CVD have also been used [6]. However, femtosecond (fs) laser writing is the only technique allowing the direct 3D microfabrication of optical waveguides [7]. Davis et al. were the first to demonstrate the modification of the refractive index using fs-laser [8]. The 3D fabrication of optical waveguides with fs-laser has great potential because it is a single-step and a mask-less process. It can also be applied to different materials (crystals [9,10], glasses [11,12] and polymers [13,14]), and the waveguide can be installed virtually anywhere within a given substrate. However, the fs-laser writing mechanism involves complex linear and non-linear properties of the material [15,16]. Moreover, important parameters such as pulse energy, repetition rate, scanning speed, number of passes, polarization, numerical aperture of the focalized beam and writing geometry need to be taken into account in the fabrication process. These parameters have been investigated in several types of glass [7,8,9,10,11,12,13,14,15,16].

In order to efficiently sense its neighboring environment via its evanescent wave, a waveguide must be located very close to the substrate surface. However, it is very difficult to write a waveguide near a surface without ablating it [17,18,19]. Waveguides laser-written at the surface can be achieved using linear absorption, which has the limitation of inscription to a plane [14]. Waveguides at the surface can also be achieved using multiscan depressed cladding waveguide or damage tracks, but this method complicates the fabrication and induces more optical losses [9]. Only a few examples of direct inscription (standard type I) of 3D waveguides near the surface have been achieved using special materials or methods. First, it was demonstrated in Gorilla glass [18], a toughen glass used for mobile device screen. An on-surface refractometric sensor for liquids was demonstrated in a smartphone screen using this approach [20]. In order to minimize the stress that causes ablation, the dual-beam technique [19] can be used to write a waveguide closer to the surface of any material. It was also demonstrated in silver-containing glass, in which the fs-laser induces the formation of silver clusters (the so-called type A waveguides) [21]. Recently, our group developed a writing technique to produce near-surface waveguides by using a compensation method [17]. By placing a glass cover slide in optical contact with the top surface of the sample, the air–glass interface is suppressed, and the fs-laser passes through unaffected. This phenomenon can be attributed to the weak Vander Waals attractive forces established between the atoms of the surfaces, which form a temporary direct bond. Therefore, it becomes possible to write waveguides at the very edge of the sample surface. After the writing process, the temporary bond is broken by immersing the assembly into water. The cover glass slide then readily lifts off, leaving the surface of the sample intact. Note that the surfaces of the sample and the cover slide must be flat and perfectly clean in order to obtain optical contact [22]. This optical contact method was adopted in the work presented in this article. Laser writing of skimming waveguides for sensing applications, which promises a high capacity for compact integration and unparalleled robustness, has raised great interest recently [23,24,25]. For a review on the subject, see Ref. [25].

In this article, we present a proof of concept of using evanescent wave coupling of buried skimming waveguides with fluorescent molecules and then with plasmonic nanostructures. We experimentally demonstrated buried waveguides at distances from the surface close enough to obtain efficient coupling with the organic fluorophore while maintaining the waveguiding properties. The photo-inscriptions were performed in two glass materials: high-purity fused silica and boro-aluminosilicate. Using silver nanoparticles organized in linear assemblies, perpendicular to the waveguide, with a wrinkled PDMS stamp technique, we demonstrated an excitation enhancement of over 20 times compared to the setup without nanoparticles. To our knowledge, this is the first time that nanoparticles are used to enhance evanescent wave coupling from an fs-laser-inscribed surface waveguide. This is of great interest for robust and compact three-dimensional integrated photonic circuits for sensing applications such as greenhouse gases detection [26], water pollutants detection [27] and biosensors [28,29].

## 2. Fabrication and Analyses of the Skimming Waveguides

The photo-inscription of near-surface waveguides was achieved according to a previously reported procedure [17] and using a modified astigmatic beam technique [30] to improve the waveguide cross section. Briefly, a femtosecond laser (Ti:sapphire, Coherent RegA) operating at a wavelength of 790 nm and a repetition rate of 250 kHz was used to modify the refractive index. The temporal FWHM of the pulses was measured to be ~70 fs at the laser output and estimated at 85 fs on the sample. The beam was focused using a 100× (Nikon, f = 2 mm, 0.8 NA) microscope objective. Samples with a good surface quality (flatness of 3λ/inch or better) were fixed on a flat mount using a temporary adhesive wax (Crystalbond 555) to ensure maximum planarity. The samples were then translated at a speed of 10 mm/s, across the focal point, perpendicular to the laser beam, using motorized mechanical stages (Newport XML210 and GTS30V). A cylindrical lens telescope and a long focal lens were used to produce an astigmatic beam and shape the focal volume in such way as to form waveguides with near-circular cross sections [30].

Very close to the surface, less than ∆*z* = 2 µm from the surface, waveguides were photo-written with a 25 mm skimming region, in the two different glass materials: a 2.5 cm × 5 cm piece of high-purity fused silica (HPFS) (Corning 7980, *n*_SiO2_ = 1.4631 at 486 nm) and a boro-aluminosilicate glass (Corning Eagle2000, *n*_Eagle_ = 1.5126 at 486 nm). The laser pulse energies used to inscribe waveguides in fused silica and boro-aluminosilicate were 0.34 µJ and 0.72 µJ, respectively. Higher laser pulse energies can induce a higher refractive index change (Δ*n*) but produce ablation near the surface [17]. These energy values were chosen to maximize the Δ*n* while allowing near-surface inscription. These glass materials are extensively used in waveguide-based photonic devices. However, borosilicate glass is a preferred material for the photo-inscription process because of its large laser pulse energy difference between the thresholds for type I modification (waveguide) and for type III modification (damage). Note that despite the narrow window for type I waveguides in fused silica, the control and enhancement of photo-induced refractive index modifications can be largely improved using a multi-scan technique [31].

To measure the photoinduced refractive index modifications, the structures were examined using a bright-field microscope (Olympus IX71) and a camera equipped with a bi-dimensional Hartmann grating (Phasics SID4Bio). The camera system acts as a wavefront analyzer that uses lateral shearing interferometry (QWLSI) to generate a quantitative phase image of transparent objects [32]. This methodology, described in detail in ref. [33], was carried out to recover the Δ*n* of the waveguides from the phase image.

**Figure 1:**
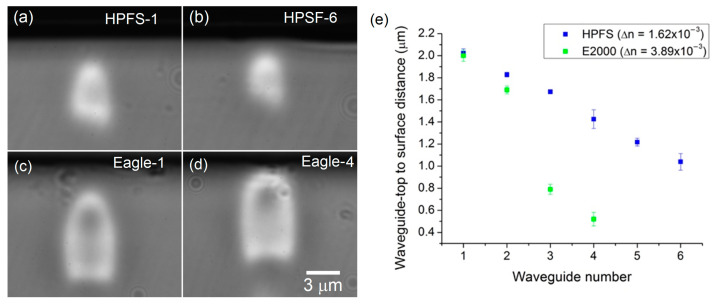
Cross section of near-surface waveguides in (**a**,**b**) fused silica, HPFS and (**c**,**d**) boro-aluminosilicate, Eagle2000. (**e**) Measured distances from waveguide-top to surface for a series (from 1 to 6) in HPFS and a series (from 1 to 4) in Eagle2000.

First, series of waveguides were inscribed in the two chosen materials. In order to study the coupling efficiency, near-surface waveguides were inscribed at the closest possible distance from the surface, i.e., before ablation. To do so, series of waveguides were made by moving the beam closer to the surface with a 200 nm increment (see Figure 1a–d). Our first observation concerned the refractive index change. Under our writing conditions, the typical ∆*n* obtained were (1.62 ± 0.2) × 10^−3^ in pure silica HPFS and (3.89 ± 0.1) × 10^−3^ in boro-aluminosilicate Eagle2000. Usually, a larger ∆*n* is preferred in photonic devices, since it allows smaller waveguide radii of curvature [7] and, thus, the fabrication of more compact devices. A large ∆*n* also produces less coupling losses from the mode mismatch with standard waveguides such as optical fibers. Distances of 0.5 µm and 1 µm were the closest-to-surface waveguides possible to write without ablation in Eagle2000 and in HPSF, respectively, for these refractive index changes, i.e., the corresponding writing conditions.

**Figure 2:**
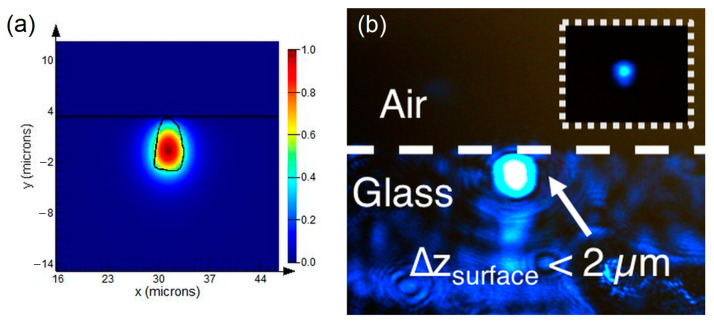
(**a**) Calculated electric E field distribution of mode propagation in an fs-laser-direct-written waveguide (the dark horizontal line is the glass/water interface, and the black contour is the waveguide structure as a reference). (**b**) Near-field intensity profile of 488 nm light observed at the output of the near-surface waveguide. Inset: attenuated near-field intensity profile.

Next, the guiding properties of these visible waveguides were analyzed by launching a pigtailed 488 nm laser diode (OZ optics, OZ-2000). Over 25% injection coupling of the 488 nm laser light was achieved using a butt coupling configuration. Losses could be mainly attributed to the mode mismatch and the misalignment of the input fiber with the waveguide glass slab. Fresnel reflections also contributed to these losses. Cut-back measurements were performed at 633 nm, and propagation losses of 1 dB/cm were measured, which is acceptable for short devices as that proposed here. The imaged near-field intensity profile of the output face of the waveguide substrate hac a nearly Gaussian intensity, revealing a single-mode propagation, as shown in Figure 2b. This experimental observation agreed with the calculated near-field mode profile (see Figure 2a). Single-mode waveguides are preferred for evanescent wave sensing applications, since low- and high-order modes have different evanescent field extents and thus different interactions with the surrounding medium [34]. In addition, for plasmonic applications, a narrower plasmon spectral band is expected with single-mode excitation, which is suitable for SPR sensing.

## 3. Fluorescence Measurements via Evanescence Field Excitation

**Figure 3:**
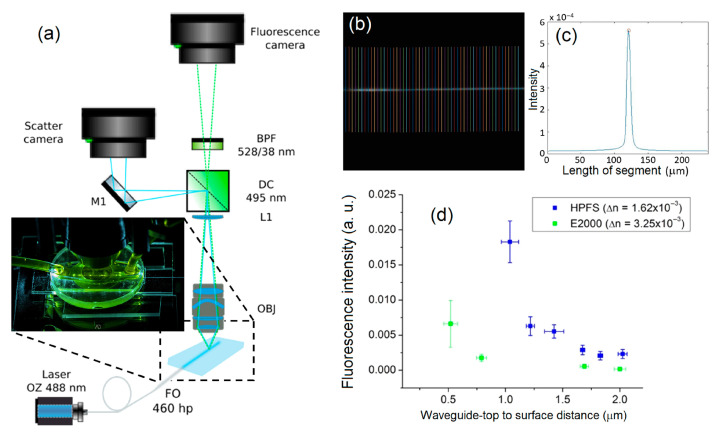
(**a**) Evanescent wave fluorescence microscopy setup. Excitation with a 488 nm laser diode injected in fiber optic (FO) (460 hp, MFD: 3.5 um, NA: 0.13). Emission measurement using an EMCCD camera with a microscope objective (Obj), a 10× lens (L1), a 495 nm dichroic filter (DC) and a 528 nm band pass filter (BPF). (**b**) Fluorescence of the skimming waveguide with an aqueous fluorescein solution and the 55 lines acquired and (**c**) the corresponding graph of one-line signal analysis. (**d**) Fluorescence sensitivity comparison between a small (∆*n* = 1.6 × 10^−3^) (blue dots with HPFS material) and a large (∆*n* = 3.2 × 10^−3^) (green dots with Eagle2000 material) refractive index change.

We investigated the evanescent coupling of these two series of skimming waveguides in the two different glass materials using fluorescent measurements. To do so, an aqueous fluorescein solution was flowed in a macrofluidic system (PDI, Bioscience tools) fixed on top of the substrate over the waveguide skimming region. Fluorescence sensitivity is a good indicator of the evanescent E field intensity reaching the substrate surface. The measurements were performed using a custom-built fluorescence microscopy setup in which these visible waveguides were used as light excitation source, as shown in Figure 3a. The skimming waveguide region in the fluorescent aqueous solution was imaged across fluorescence filters on an EMCCD camera (Princeton Instruments, PhotonMax512). For each waveguide image, 55 acquisition lines (added in Figure 3b for illustrative purpose) crossing perpendicularly the skimming waveguide region were used to measure the fluorescence signal. Using Matlab, the fluorescence intensity value was obtained by averaging the 55 maxima (see Figure 3c). Figure 3c shows the high signal-to-background ratio toward the waveguide resulting from using a near-field excitation configuration technique.

As shown in Figure 3d, starting from the most embedded waveguides (HPFS-1 and Eagle2000-1), the sensitivity increased exponentially up to the closest waveguides (HPFS-6 and Eagle2000-4). While writing near-surface waveguides, the laser focal spot was close to the interface, and the ablation process occurred easily at this location despite the compensation method used. The energy threshold for ablation was lower in fused silica than in alumino-borosilicate. Therefore, the waveguides in Eagle2000 could reach a closer distance from the surface (Figure 1e). In order to write waveguides at a similar distance in HPFS, the laser inscription power had to be decreased, with the drawback of a reduced refractive index change. However, this moderate refractive index change is interesting for evanescent wave sensing, since the numerical aperture of optical waveguides is proportional to the refractive index difference between the core and the cladding. This is reflected in our fluorescence sensitivity experiments. Even if some waveguides of Eagle2000 were closer to the surface, there was a significant decrease in fluorescence sensitivity (Figure 3d). The evanescent wave exponential decay was indeed different for these two series of waveguides. The decay for the Eagle2000 was faster than that for the HPFS because of the larger ∆*n*, which confined the evanescent *E* field. Thus, and as expected, for a fixed distance from the surface, we observed a weaker sensing performance when using a larger ∆*n*.

## 4. Enhancing Evanescent Wave Coupling Using Nanoparticles

The following section presents a novel method to enhance the evanescent coupling of near-surface waveguides using a plasmonic layer made of silver sphere nanoparticles placed on the substrate surface. The localized surface plasmon resonance (LSPR) of silver sphere nanoparticles and their larger absorption cross section with respect to molecules result in a metal-enhanced fluorescence (MEF) [35]. In order to confirm the MEF effect and visualize it, we first tried to couple the evanescent field of our less sensitive near-surface waveguides, which were in Eagle2000, with silver plasmonic nanoparticles in suspension. These plasmonic nanoparticles were synthesized according to a seed-growth procedure [36]. A colloidal suspension of silver particles with a final 80 nm diameter was used with maximal LSPR plasmonic absorption properties at 480 nm. A solution with silver particles in suspension with a concentration of 10^9^ nanoparticles/mL was flowed over the skimming waveguide region. As expected, we observed that the majority of the scattering signal was coming from particles over the waveguide and interacting with the evanescent field.

In order to increase the signal, a more concentrated suspension of Ag nanoparticles was organized in linear assemblies with a wrinkled PDMS stamp technique [37]. This inexpensive fabrication technique does not require highly sophisticated equipment and can produce a large number of linear assemblies on surfaces up to several mm^2^. In order to reproduce our work, here is a brief summary of the technique as well as the manufacturing parameters used.

Using a cross-linked polymer such as PDMS, it is possible to form a thin, rigid layer on its surface called the “skin.” This skin can be formed through chemical oxidation using an oxidizing gas or by depositing a metallic layer. Thus, when this rigid layer is deposited onto a polymer subjected to mechanical strain, such as stretching, the polymer will form periodic wrinkle structures during relaxation. These wrinkles result from an incompatibility of the elasticity properties (Young’s modulus) between the skin and the non-oxidized polymeric film, with the applied strain, in this case stretching, dictating the orientation of the formed wrinkles.

**Figure 4:**
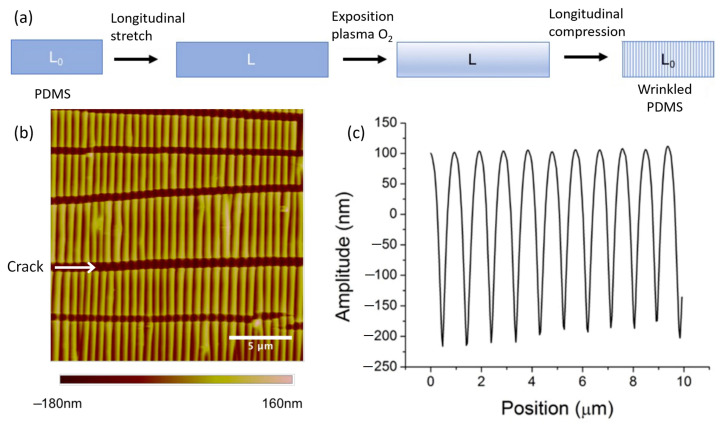
(**a**) Process of creating wrinkles on the surface of PDMS involved applying longitudinal stretching before an oxygen plasma treatment. The wrinkles formed when the PDMS substrate relaxed. (**b**) AFM image of a PDMS substrate showing perpendicular cracks to the linear wrinkles. These cracks are oriented in the same direction as the applied stretching. (**c**) Typical profile of the wrinkles in (**b**).

In order for the formation of wrinkles to be permanent, the stress must be applied before the formation of the rigid layer. Thus, upon relaxation of this stretching stress, a compressive stress will fix the wrinkles in the absence of stretching (see Figure 4a). The orientation of the wrinkles is perpendicular to the direction of the stress. The formation of a thin rigid layer was carried out by an oxidizing treatment using oxygen plasma. This process was chosen based on the range of wrinkle pitches obtained using different approaches. Indeed, as the goal of this work was to study the optical properties of linear assemblies of plasmonic nanoparticles, the modulation of the dimensions at the nanometer scale was necessary.

The morphology of the formed wrinkles was analyzed by atomic force microscopy (AFM), as shown in Figure 4b. The typical profile of the wrinkles is shown in Figure 4c. First, it can be observed that the formed wrinkles had a quasi-sinusoidal shape, a micrometer pitch and a nanometer-scale amplitude (or depth). The morphology and size of the wrinkles were very regular, which is very useful for the fabrication of organized particle substrates on a millimeter scale. However, these linear structures are not without defects. Indeed, the use of a plasma treatment induces cracks perpendicular to the wrinkles due to the Poisson effect that occurs during the contraction of the material perpendicular to the direction of the applied force (see white arrow in Figure 4b). It is possible to minimize the formation of these cracks by reducing the thickness of the rigid layer through a decrease in exposure duration or plasma furnace pressure.

**Figure 5:**
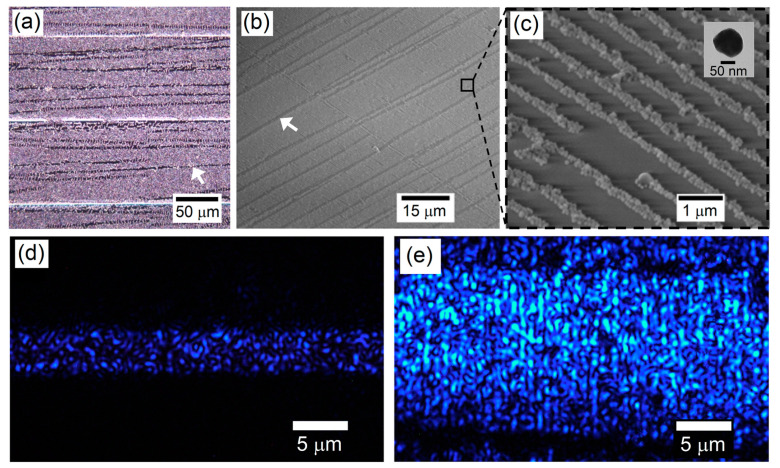
(**a**) Dark-field microscopy images of a linear array of Ag nanoparticles on the Eagle2000 waveguide substrate. The white horizontal lines are ablated regions corresponding to damaged waveguides (for reference, not used in the experiment). The skimming waveguides (not visible on the image) are between and parallel to the ablated lines. (**b**,**c**) SEM images of the linear array. The inset in (**c**) is a TEM image showing the typical shape of a single Ag nanoparticle used in this work. The linear array of nanoparticles can be seen using SEM, while it cannot be seen in (**a**). The two white arrows in (**a**,**b**) point to a boundary line between two linear assemblies of nanoparticles, produced by the cracks in the wrinkled PDMS. (**d**) Waveguide scattering without the nanoparticles. (**e**) Waveguide scattering with the linear array of Ag nanoparticles.

The influence of various parameters on the size of the wrinkles formed on PDMS was investigated in order to determine the optimal conditions for the linear assembly of metal nanoparticles. Several experimental parameters, such as cooking temperature and time to increase PDMS Young’s modulus (tested from 40 °C to 80 °C for 2 to 5 h), plasma exposure time (tested from 25 s to 240 s), plasma oven pressure (tested from 6 to 10 Torr) and stretching force applied on PDMS (tested from 15% to 40% stretching), were studied. The investigation of these parameters allowed the determination of ideal conditions for the assembly of plasmonic core–shell particles. In general, we observed that very small wrinkles could be manufactured at high cooking temperatures and times, short plasma exposure times, low stretching percentages, and low pressures in the plasma oven. We also found that a second plasma treatment without applying a stretching force to the wrinkled PDMS could slightly reduce the depth of the wrinkles without affecting their period. In this project, nanoparticle assembly was carried out under the following optimal conditions: 1. use 15% stretching of PDMS cooked at 80 °C for 5 h; 2. apply an O_2_ oxidizing treatment under 6 Torr for 60 s; 3. apply a second oxidizing treatment of 120 s to make the surface as hydrophilic as possible. These parameters proved to be optimal for aligning lines of Ag nanoparticles of various diameters ranging from 30 to 100 nm. In order to arrange the nanoparticles in a linear array on the waveguide substrate, a droplet of nanoparticle solution must be deposited between the PDMS and the clean substrate and allowed to dry for one hour without applying pressure to the substrate. To ensure a homogenous array, it is preferable to centrifuge the solution to increase the nanoparticle concentration. Once the substrate is dry, it can be tilted, causing the PDMS layer to detach. A successful linear assembly is indicated by the formation of a diffraction grating on the substrate, which manifests as a rainbow effect. The substrate can be preserved in a dark environment for several weeks. These linear assemblies produce an improved surface coverage of particles over the skimming waveguide substrate (see Figure 5a–c). It has also been demonstrated that small inter-particle gaps in linear assemblies create plasmonic hotspots for enhanced fluorescence excitation [38]. Adding this plasmonic array perpendicular to the waveguide will result in a much more intense scattering and an excitation section whose spatial dimensions exceed that of the waveguide itself, as shown in Figure 5e. Note that the effect of the cracks in the wrinkled PDMS (see white arrow in Figure 4b) can be observed in the images of the linear array of nanoparticles. The two white arrows in Figure 5a and b indicate examples of the boundary line produced by a crack located between two linear assemblies of nanoparticles. Special care must be taken when stamping the PDMS on a glass substrate, so that these cracks do not overlap with the surface-skimming waveguides, especially when only a single waveguide is inscribed.

**Figure 6:**
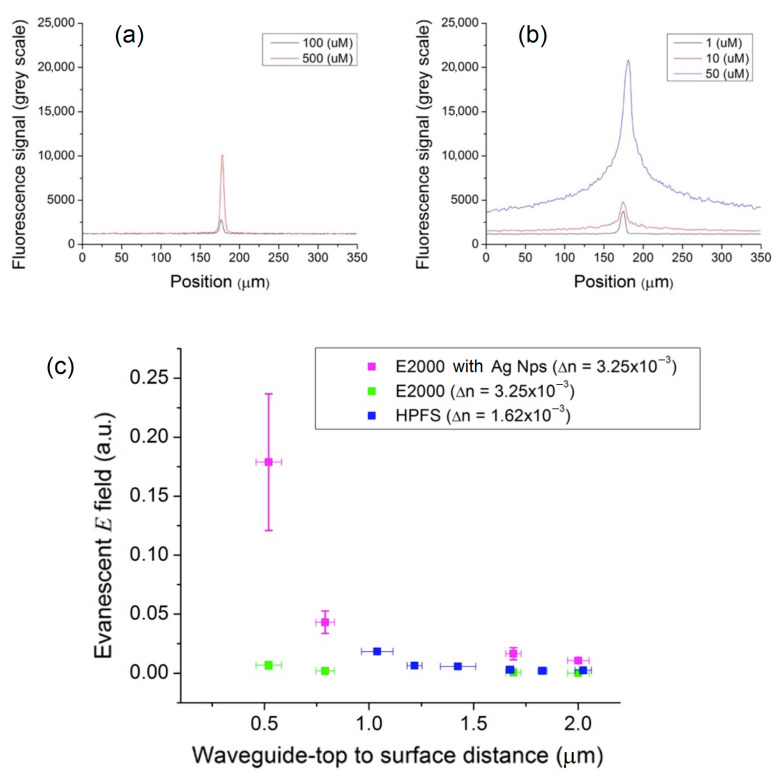
Signal-to-background ratio comparison of perpendicular acquisition line profiles across fluorescence images on the Eagle2000 glass waveguide (**a**) without Ag nanoparticles and (**b**) with the Ag nanoparticle array. (**c**) Fluorescence sensitivities comparison between HPFS, Eagle2000 and Eagle2000 with the plasmonic Ag-80 nm nanoparticle linear array.

Subsequently, fluorescence measurements were carried out with the same waveguides in the Eagle2000 borosilicate substrate, but in the presence of the plasmonic array in order to evaluate the magnitude of MEF exaltation. Identical methodology and experimental setup as described in detail in Section 3 were employed. As shown in Figure 6a,b, in the absence of the plasmonic array, the ratio of the fluorescence signal to the background signal or the signal-to-background ratio (SBR) was 2.2 and 8.3 for fluorescein solutions at the respective concentrations of 100 μM and 500 μM. As expected, the addition of the plasmonic array greatly increased the measured fluorescence signal, and very similar SBR ratios, i.e., 3.0 and 5.5 for the 10 µM and 50 µM solutions, respectively, were calculated. Note the plasmonic enhancement effect allowing fluorescence reading for solutions whose fluorophore concentration was an order of magnitude lower.

An average fluorescence sensitivity enhancement factor of 27 ± 3 was obtained when the plasmonic array was added to the surface of high-index waveguides in Eagle2000 (see Figure 6c). One can clearly observe the effect of fluorescence enhancement by metals, particularly for the guide closest to the surface. This MEF enhancement was attributed to the strong absorption cross section of the evanescent field by the plasmonic particles and to the re-scattering of the energy captured by the particles towards the fluorescent solution.

Future works will include testing the coupling between two adjacent surface-skimming waveguides via the strong scattering surface induced by the Ag nanoparticle linear assemblies. Indeed, using a single skimming waveguide, only part of the guided light interacted with the nanoparticles via the evanescent field. Most of the light measured at the waveguide output, therefore, did not interact with the nanoparticles, which makes the sensor much less sensitive to the external environment. Using a second adjacent skimming waveguide, only the light that interacts with the nanoparticles will be coupled to the second skimming waveguide. We expect that by using two adjacent surface-skimming waveguides along with the Ag nanoparticle linear assemblies, the sensitivity of the sensor should increase significantly.

## 5. Conclusions

In summary, our study successfully demonstrated the fabrication of surface-skimming waveguides using pure silica (HPFS) and boro-aluminosilicate (Eagle2000), with reproducible and tunable refractive index changes and surface-to-waveguide distances. We were able to achieve an efficient evanescent field coupling with dye-sensitive molecules by reducing the waveguide-to-surface distance to approximately 1 µm. Additionally, our study demonstrated a reduction in sensing efficiency for waveguides written at their minimum distance to the surface without ablation, as the refractive index contrast of the waveguide increased. While this result was anticipated, it had not been previously demonstrated in the literature. We also found that Eagle2000 glass is suitable for designing near-surface optical waveguides sensors because of the large laser pulse energy difference between the thresholds for refractive index modification and for ablation. Furthermore, a V-number of about 2.5 was measured in the waveguides with a small ∆*n* inscribed in HPFS, resulting in single-mode operation that is suitable for angular-dependent SPR applications. Finally, using an evanescent wave fluorescence microscopy setup, we observed a stronger evanescent coupling with plasmonic silver nanoparticles placed on the surface-skimming waveguides. Using silver nanoparticles organized in linear assemblies, perpendicular to the waveguide, with a wrinkled PDMS stamp technique, we demonstrated an excitation enhancement of over 20 times compared to the setup without nanoparticles. Our results pave the way for the fabrication of novel integrated photonic devices based on dielectric-to-plasmonic coupling and photoexcitation of on-surface nanostructures. Furthermore, the linear array of plasmonic nanoparticles could potentially be used for energy transport between adjacent waveguides, thus allowing to significantly improve the signal-to-noise ratio of surface sensors. Overall, our findings provide valuable insights for advancing surface-sensing applications and integrated photonics research.

## Data Availability

Not applicable.
